# Maine organic dairy producers’ receptiveness to seaweed supplementation and effect of *Chondrus crispus* on enteric methane emissions in lactating cows

**DOI:** 10.3389/fvets.2023.1153097

**Published:** 2023-07-06

**Authors:** Diana C. Reyes, Jennifer Meredith, Leah Puro, Katherine Berry, Richard Kersbergen, Kathy J. Soder, Charlotte Quigley, Michael Donihue, Dorn Cox, Nichole N. Price, Andre F. Brito

**Affiliations:** ^1^Department of Agriculture, Nutrition, and Food Systems, University of New Hampshire, Durham, NH, United States; ^2^Department of Economics, Colby College, Waterville, ME, United States; ^3^Wolfe’s Neck Center for the Agriculture and the Environment, Freeport, ME, United States; ^4^Cooperative Extension, University of Maine, Orono, ME, United States; ^5^USDA-ARS, Pasture Systems and Watershed Management Research Unit, University Park, PA, United States; ^6^Bigelow Laboratory for Ocean Sciences, East Boothbay, ME, United States

**Keywords:** alternative feed, greenhouse gases, milk quality, mitigation, survey

## Abstract

**Introduction:**

There is a growing interest in utilizing seaweed in ruminant diets for mitigating enteric methane (CH_4_) emissions while improving animal health. *Chondrus crispus* is a red seaweed that grows in the Gulf of Maine (United States) and has shown to suppress CH_4_ production *in vitro*. Organic dairy producers in Maine are currently feeding seaweed due to herd health promoting benefits. However, large-scale adoption depends on technical and financial factors, as well as validation from pilot studies.

**Methods:**

A survey was developed to identify barriers and drivers towards the adoption of CH_4_-reducing algal-based feeds. Concurrently, a randomized complete block design study was conducted to investigate the effect of *C. crispus* on enteric CH_4_ emissions and milk production in a typical Maine organic dairy farm. Twenty-two organically certified Holstein and Jersey cows averaging 29 ± 6.8 kg of milk/d and 150 ± 69 days in milk, were blocked and randomly assigned to a control diet without *C. crispus* (0CC), or with 6% [dry matter (DM) basis] *C. crispus* (6CC). Samples were collected on the last week of the 2-wk covariate period, and wk 3, 5, 8, and 10 after initiation of treatments for a total of 12 weeks. Gaseous emissions were measured using a GreenFeed unit. Data were analyzed using the MIXED procedure of SAS with repeated measures over time.

**Results:**

All survey respondents (*n* = 35; 54% response rate) were familiar with seaweeds as feed, and 34% were already users. Producers who were willing to pay 0.64 USD/cow/d on average for a CH_4_-reducing algal-based feed, also stated the need for co-benefits in terms of cattle health and performance as a requirement for adoption. Feeding 6CC decreased enteric CH_4_ production by 13.9% compared with 0CC (401 vs. 466 g/d). Further, milk yield (mean = 27.1 kg/d), CH_4_ intensity (mean = 15.2 g of CH_4_/kg of energy corrected milk), and concentrations and yields of milk fat and true protein were not affected by treatments.

**Discussion:**

Producer receptiveness to CH_4_-reducing algal-based feeds will not only be dependent on purchase price, but also on co-benefits and simplicity of integration into existing feed practices. Feeding *C. crispus* at 6% of the diet DM decreased CH_4_ production in dairy cows by 13.9% without negative effects on milk yield and composition. Identifying the bioactive compounds in *C. crispus* is critical to understand the effect of this red seaweed on mitigating enteric CH_4_ emissions in dairy cows.

## 1. Introduction

The dairy industry is under increasing scrutiny regarding its role in global greenhouse gas emissions, mainly from enteric methane (CH_4_). There is a growing interest in incorporating algal-based feeds in ruminant diets motivated by their potential for reducing enteric CH_4_ emissions while improving animal health (1–3). Seaweed contains secondary compounds with broad bioactive properties, some well-known for their antimethanogenic activity [e.g., bromoform, phlorotannins (4)]. However, factors such as seaweed species, geographical location, processing, and dietary inclusion rates may affect the extent of CH_4_ suppression when feeding algae (5). The red seaweeds *Asparagopsis taxiformis* and *Asparagopsis armata* have been identified for their high antimethanogenic activity, decreasing CH_4_ production in sheep (6), dairy cows (2, 7), and beef cattle up to 80% (8). The mechanism of inhibition has been correlated to the accumulation of halogenated compounds such as bromoform (9). Nonetheless, these seaweeds do not grow in colder climates, are highly invasive, and yield low biomass (10). Therefore, scaling up production to be used in commercial dairy operations is challenging. Moreover, concerns have been raised related to high and variable iodine concentration in *Asparagopsis* species which may be transferred to milk and must be considered in future research (11). Currently, the brown seaweed *Ascophyllum nodosum*, commercialized as kelp meal, is the most popular algal-based feed used in organic dairy farms in the United States (12–15). A recent survey revealed that 72.5% of the organic grass-fed dairy producers in the United States feed *A. nodosum* (15). Perceived benefits reported by producers in response to *A. nodosum* supplementation include improved body condition and overall animal appearance, as well as decreased reproductive problems, milk somatic cell count, and incidence of infectious bovine keratoconjunctivitis [i.e., pinkeye (12)]. However, feeding *A. nodosum* meal to organic dairy cows during the grazing season had minor effects on suppressing enteric CH_4_ emissions (16).

Supplementing an algal-based feed that would provide the benefit of reducing the impact of greenhouse gas emissions from farms may be appealing to organic dairy producers if animal production and health are not compromised. *Chondrus crispus* is a red seaweed that is wild harvested from the intertidal zone of the North Atlantic, including the Gulf of Maine (United States), that has exhibited potential to suppress CH_4_ production *in vitro,* and is available under organic certification. Results from a study conducted at the University of New Hampshire in which incremental amounts [0, 3, and 6%; diet dry matter (DM) basis] of *C. crispus* were fed to organic dairy cows given a baleage-based diet, showed a linear decrease in enteric CH_4_ production, with the greatest dietary level resulting in an 8.4% reduction in CH_4_ compared with the control diet (17). However, further research is needed to validate the effect of *C. crispus* on enteric CH_4_ emissions and production performance under different production systems. Moreover, beyond scientific validation, diverse technical, financial, and social factors may dictate the receptiveness of dairy producers towards algal-based feeds. Therefore, research that incorporates the concerns of stakeholders and the marketability of seaweed products are increasingly necessary (18, 19). Our first objective was to quantitatively survey the willingness of organic dairy producers in Maine (United States) to adopt an algal-based feed that decreases enteric CH_4_ emissions. Our second objective was to conduct an animal case study feeding trial to investigate the effect of *C. crispus* on enteric CH_4_ emissions and production performance in lactating dairy cows from a herd representative of a typical organic dairy farm of the state of Maine.

## 2. Materials and methods

### 2.1. Survey development and administration

The survey was developed with the aim of determining Maine organic dairy producers’ perception and willingness for adoption of an algal-based feed that would decrease enteric CH_4_ emissions. The survey was approved by the Colby College Institutional Review Board (Approval Number 2021-099). The survey was first announced to 65 organic dairy producers through a flyer (Appendix A), followed by the survey instrument, which was conducted telephonically by interviewers from Colby College (Waterville, Maine) between March and June 2021. The questionnaire (Appendix B) included the following sections: (1) general farm and herd production descriptors, (2) feeding and management practices that could potentially contribute to the design of an effective algal-based feed, and (3) current seaweed supplementation practices and their willingness to adopt and pay for a hypothetical supplement that had been proven to reduce enteric CH_4_ emissions. Survey data were described with summary statistics including means and standard deviations (Appendix C). Data were analyzed using Stata (version 17, StataCorp LLC).

### 2.2. Animal case study feeding trial

An animal case study feeding trial examining the effect of the red seaweed *C. crispus* on enteric CH_4_ emissions and milk production and composition in dairy cows was conducted at Wolfe’s Neck Center for Agriculture and the Environment. This is a 243-hectare certified organic and preserved coastal farm located in Freeport, Maine (43°49′50.7′N, 70°04′17′W), with an educational and regenerative farming mission. At the time of the study, the organic dairy herd consisted of 32 lactating dairy cows (Holstein, Jersey, and crossbred) producing an average of 27.4 kg milk/d, with 22 animals selected to participate in the study. All experimental procedures were reviewed and approved by the University of New Hampshire Institutional Animal Care and Use Committee (IACUC protocol # 200502).

#### 2.2.1. Cows, experimental design, and treatments

Eighteen Holstein and 4 Jersey cows (averaging 144 ± 72 and 175 ± 53 days in milk in the beginning of the study, respectively), were used in a 12-wk randomized complete block design study conducted from February to May 2021. Cows were blocked (*n* = 4 blocks) according to breed and days in milk, and within block, randomly assigned to a control diet without *C. crispus* supplementation (0CC) or a diet containing (DM) basis 6% *C. crispus* (6CC). The seaweed (Wild North Atlantic Irish Moss; Vitamin Sea, Scarborough, Maine) was harvested in the summer of 2020, air-dried indoors for 24 to 72 h, by spreading it over a plastic film, frequently turning it to remove moisture, and finally milled to pass through a 3-mm screen (small flake size). Cows were housed in a compost-bedded pack barn with dried pine shavings as bedding, and milked twice daily at 0530 and 1630 h, with milk yield recorded throughout the experiment using electronic milkmeters (SCR, Madison, WI). Diets were formulated with a forage to concentrate ratio (% of diet DM) of 67:33 with each component of the ration fed separately. The forage component of the diet was a mixture of chopped, first and second cut grass-legume baleage mix (mostly grass) and alfalfa baleages. A ground corn-based concentrate pellet made up the remainder of the diet. The seaweed was mixed by hand with the concentrate and fed 4 times/d (before and after the morning and afternoon milkings) individually when cows were restrained in headlocks for approximately 20 min to ensure complete seaweed consumption.

#### 2.2.2. Sampling and analyses

Milk and gaseous samples were taken on the last week of the 2-week covariate period, and week 3, 5, 8, and 10 after treatments began. Body weight (BW) was measured for 3 consecutive days before the beginning of the experiment and at the end of each sampling week with a SmartScale (C-Lock Inc., Rapid City, SD). Estimated DM intake (DMI) was determined using the Nutrient Requirements of Dairy Cattle (2001) equation:


$$\begin{array}{c} DMI\;\left(kg/d\right)=\left(0.372\times FCM+0.0968\times BW^{0.75}\right)\\ \times \left\{1-e^{\left[-0.192\times \left(WOL+3.67\right)\right]}\right\} \end{array}$$

where FCM = 4% fat corrected milk (kg/d), BW^0.75^ is metabolic BW in kg, and WOL = week of lactation (20). Feed samples (forage, concentrate, and seaweed) were collected weekly, composited by month, and analyzed for DM, crude protein (CP), neutral detergent fiber, acid detergent fiber, acid detergent lignin, ether extract, ash, and minerals, following wet chemistry procedures used by Dairy One Forage Laboratory [Ithaca, NY; (21)]. The iodine concentration of *C. crispus* was analyzed via quadrupole inductively coupled plasma mass spectrometry at the Bigelow Laboratory for Ocean Sciences, Trace Metal Biogeochemistry Laboratory (East Boothbay, ME). The presence of bromoform and other brominated compounds (including dibromomethane) in *C. crispus* was analyzed by Bigelow Analytical Services (East Boothbay, ME). Samples of *C. crispus* were extracted in methanol using a bead-beating protocol. Fifty to 200 mg of sample was weighed out into 1.5-mL microcentrifuge tubes containing ~0.8 g of 2.0 mm Zirconia Oxide beads. One mL of methanol extractant containing the internal standard (naphthalene) was added and the samples were bead-beat for 15 min at 30 Hz. The tubes were then centrifuged at ~21,000 g for 5 min and the supernatant transferred to autosampler vials and analyzed on a Shimadzu GCMS-QP2010 (Shimadzu Scientific Instruments Inc., Columbia, MD). The column used was a 30 m × 0.25 mm × 1.4 μm film thickness Restek Rtx 502.2 (Restek Corporation U.S., Bellefonte PA). The injection temperature was 200°C, and the ion source and interface temperatures were both 220°C. The GC–MS column oven temperature was programmed to start at 40°C for 4 min, increase at a rate of 15°C to 80°C, increase at 30°C to a final temperature of 200°C, and hold for 5 min. The injection volume was 5 μL. Helium was used as carrier gas at a constant pressure of 10.5 psi, giving a column flow rate of 1.13 mL/min. For quantification, the major fragment ions were mass/charge ratio (m/z) 173 and 128 for bromoform and naphthalene, respectively. The limit of detection was 0.012 μg bromoform/g of seaweed (dry weight).

Milk samples were collected during 4 consecutive milkings starting on Monday afternoon of each sampling week, composited based on individual milk yield, preserved with a 2-bromo-2-nitropropane-1,3 diol tablet, and refrigerated at 4°C until analyses. Composited milk samples were shipped to Dairy One Laboratory (Ithaca, NY) and analyzed for concentrations of fat, true protein, and milk urea N (MUN) by Fourier transform infrared spectroscopy using a MilkoScan model 6,000 (Foss Inc., Hillerød, Denmark). Production of 4% FCM and energy corrected milk (ECM) were calculated using the equations reported by Gaines and Davidson (22) and Tyrrell and Reid (23), respectively. Cows had access to a portable automated open-circuit gas quantification unit (i.e., GreenFeed system, C-Lock Inc., Rapid City, SD) for gaseous measurements [CH_4_ and carbon dioxide (CO_2_)] throughout the experiment. GreenFeed calibrations were performed weekly using a zero [nitrogen (N_2_); baseline gas] and a span gas mixture (CO_2_ and CH_4_) as outlined by Hristov et al. (24), and a CO_2_ recovery test was conducted once a month, with a mean (±SD) recovery of 103 ± 3.9%. The same ground corn-based concentrate pellet from the diet (4.4 mm diameter) was used as a bait to attract cows to the unit. In 24 h, cows were allowed a maximum of 6 visits of up to 6 min each (average visit time = 05:24 min), with 4 h intervals between visits, and no more than 12 pellet drops of 33 ± 1.05 g (as fed) per visit.

#### 2.2.3. Statistical analysis

Data (excluding estimated DMI) were analyzed for a randomized complete block design with repeated measures over time using the MIXED procedure of SAS (version 9.4, SAS Institute Inc., Carey, NC). Yields of milk and concentrations and yields of milk components, and gaseous measurements were used as covariate variables in the statistical model reported below:


$$Y_{ijkl}=\mu +B_{i}+T_{j}+W_{k}+\beta C_{ijkl}+T\times W_{jk}+\varepsilon_{ijkl}$$

where Y_ijkl_ = dependent variable, μ = overall mean, B_i_ = random effect of the ith block, T_j_ = fixed effect of the jth treatment, W_k_ = fixed effect of the kth sampling week, β = the regression coefficient of the covariate C, C_ijkl_ = the covariate variable for the lth cow within the ith block of the jth treatment of in the kth week, T × W_jk_ = interaction between the jth treatment and the kth week, and ε_ijkl_ = residual error. The SAS command REPEATED was used for modeling distinct residual variances, and the covariance structure with the lowest Bayesian information criterion value was retained in the final model. The following covariance matrices were tested: variance components, spatial power, compound symmetry, autoregressive (1), and heterogeneous autoregressive (1). Cow nested within treatment was defined as the subject of the repeated measures and treated as a random effect in the model. Least square means were separated by pairwise *t*-test using the PDIFF option of the MIXED procedure of SAS if *p* ≤ 0.05. Furthermore, least square means within sampling week were partitioned with the SLICE command of SAS and separated by pairwise *t*-test when diet × week interactions were *p* ≤ 0.05. Significance was declared at *p* ≤ 0.05 and tendencies at 0.05 < *p* ≤ 0.10.

## 3. Results and discussion

### 3.1. Survey

In total, 35 organic dairy producers participated in the survey (including the Wolfe’s Neck Center for Agriculture and the Environment dairy farm), yielding a response rate of 54%. The organic dairy sector in Maine is diverse (mean ± standard deviation; Appendix C, Supplementary Table C1) in terms of herd size (53 ± 47 lactating cows), productivity (21.1 ± 6.98 kg milk/cow/d), and milk contract price (0.68 ± 0.15 USD/kg of milk). Similarly, dietary inclusion of pasture is highly variable (65.3 ± 21.7%) and it was a key determinant of producer receptiveness to the algal-based feed as discussed below. Nearly half of the respondents (48.6%) reported willingness to pay on average 0.64 USD (± 1.33) per cow daily for an algal-based feed that had been proven to reduce enteric CH_4_ emissions (Appendix C, Supplementary Table C1). This would represent an additional feed cost of 4,523.38 USD per year to the average farm (53 lactating cows). It is noteworthy to mention that the self-reported price producers were willing to pay (0.64 USD/cow/d) may have been inflated by hypothetical survey bias and further research investigating consumer and producer preference is needed before the marketability of a supplement can be determined. The producers that were willing to pay for an algal-based feed were characterized by having 16% less dietary inclusion of pasture and 10% greater milk contract price (*p* < 0.05; Table 1). These differences indicate that the best predictors of voluntary supplement demand within the market are farm revenue and convenience of delivery. On the other hand, farm size was not significantly correlated with willingness to pay or with an increased value for the hypothetical supplement (*p* = 0.34).

**Table 1:** Maine organic dairy producer differences in willingness to pay for an enteric methane-reducing seaweed supplement^1^^,^^2^.

Variable	Willing	Unwilling	Difference (%)
Number of farms in survey	12	23	–
Cows per herd	60	46	0.23
Milk yield, kg/cow/d	20.5	21.9	−0.07
Milk fat, %	4.29	4.38	−0.02
Milk true protein, %	3.27	3.71	−0.13
Milk somatic cell count, ×10^3^ cells/mL	125.1	121.4	0.03
Milk shipped, kg/year	435,800	548,771	−0.26
Contract price, USD/cwt of milk^3^	32.6	29.3	0.10^*^
Pasture in the diet, %	61.1	70.6	−0.16^**^
Grain cost, USD/ton	660.9	691.4	−0.05

1 Willingness to pay measured as response to survey question “How much of an increase in your daily feed costs would you be willing to pay for a seaweed supplement that also reduced methane emissions?” Those producers who indicated they would be willing to pay any positive amount are included in the mean for those willing to pay. The producers who indicated they would not be willing to pay anything for the supplement are in the mean for those unwilling to pay.

2 Difference measured as the average of those willing to pay minus the average of those unwilling to pay and reported in percentage terms. **difference is significant with 5% confidence, *difference is significant with 10% confidence.

3 Cwt = milk price received per hundredweight (cwt = 45.36 kg).

All respondents were familiar with algal-based feeds, and 34% were already feeding them (i.e., *A. nodosum* meal) to their herds, and were paying an average of 0.45 USD/cow/d. They reported diverse perceived benefits such as improved reproductive health, provision of micronutrients, and decreased incidence of pink eye and ringworm, thus in line with Antaya et al. (12), who reported that 58% of US northeastern organic dairy producers feed *A. nodosum* meal due to decreased reproductive problems, milk somatic cells count, and pink eye incidence. Of the 23 producers who were no longer feeding seaweed, 7 had discontinued use due to price increase, 2 because of complications obtaining and storing these feeds, and 4 due to a lack of perceived benefits.

Barriers for implementing the hypothetical CH_4_-reducing algal based feed are presented in Table 2. Beyond the price of the supplement, organic dairy producers who reported qualifications for their willingness to adopt were most likely to cite the existence of co-benefits such as improved milk production as a precondition for their adoption. One respondent expressed “Just using for CH_4_ production is a strong maybe, but if it helped with milk production and other factors, we would make sure to at least consider it.” Lastly, of the 6 who were unambiguously unwilling to adopt the hypothetical supplement, 5 expressed skepticisms about the link between dairy CH_4_ emissions and climate change and 1 was concerned that the scale of their farm was too small to make a difference.

**Table 2:** Qualifications to Maine organic dairy producers’ willingness to adopt^1^ a methane-reducing seaweed supplement.

Response *Willing to adopt if…*	Frequency^2^
There were benefits beyond methane	8
It is cost-effective	6
It specifically improves cattle health	2
It specifically improves milk production	2
The price and nutrients were equivalent to current kelp supplements	1
It helped attain CROPP^3^ climate goals	1

1 Willingness to adopt measured as response to survey question “If different types of seaweed supplements become available that would reduce the amount of methane emissions from your cows would you be interested in adding it to their feed?”

2 Of the 35 producers asked this question, 6 unambiguously said no, 12 unambiguously said yes, and 17 gave qualifications to their response. The number of producers reporting a given response is from this group of 17. Producer responses can fall into multiple categories.

3 CROPP refers to the Organic Valley farmer-owned cooperative.

Producers were also asked about the top 3 challenges they face as organic dairy producers in Maine. More than 54% listed rising input costs as a primary concern, with labor shortages, failing infrastructure, and supply chain issues also being listed by more than a third of the sample. Conversely, only 4 producers cited a changing climate or climate regulations in their list. Companies within the dairy processing sector anticipate stricter climate regulations and are voluntarily making corporate social responsibility commitments to proactively lower their climate footprint (25). However, organic dairy producers are already operating at low profit margins and seem willing to prioritize CH_4_-reducing innovations only when mandated by their contracted milk buyers, when the animal health co-benefits compensate them for the additional expense, or when climate-conscious consumers are willing to pay a premium for their product.

### 3.2. Animal case study feeding trial

The ingredient and nutritional composition of the experimental diets are presented in Table 3. The ingredient composition of the concentrate pellet is shown in Table 4, and the nutritional composition of *C. crispus* and other dietary ingredients is presented in Table 5. Iodine concentration is typically greater in brown versus red seaweeds (26). However, lower iodine values were reported for *A. nodosum* [mean = 654 ± 212 mg/kg of DM (12, 16, 27)] relative to *A. taxiformis* [2,270 mg/kg of DM (8)]. This discrepancy may be attributed to intrinsic species differences, effects of seasonality, geographical location, and harvesting and processing methods (28). Considering that iodine can be transferred from feed into milk, the high concentration of iodine in seaweeds in studies conducted with lactating dairy cows, have raised animal and human-health concerns (29). Although high iodine intake is well tolerated by most healthy individuals, people with underlying thyroid conditions or susceptible groups (e.g., elderly and neonates), may develop goiter (thyroid enlargement), hyperthyroidism, or hypothyroidism (30, 31). In the United States, the recommended dietary allowance for adults is 150 μg/d, and the tolerable upper intake level is 1,100 μg/d (32). According to the European Food Safety Authority (33), milk iodine concentrations should remain below 500 μg/L to minimize toxicity risks. However, as recently summarized by Brito (34), milk iodine concentrations above the 500-μg/L threshold have been reported in response to dietary supplementation of *A. nodosum* (12, 27), *A. taxiformis* (2), and *C. crispus* (35) to dairy cows. Consequently, it is imperative to develop technologies that enable dairy producers to feed safe and predictable algal-based feeds to decrease the risk of excess iodine intake by cows and humans.

**Table 3:** Ingredient and nutritional composition [% of diet dry matter (DM)] of diets with *Chondrus crispus* at 6% DM (6CC) or without (0CC).

	Treatment	
Item	0CC	6CC
**Ingredient, % of the diet DM**		
Concentrate pellet	33.2	32.2
Alfalfa baleage	24.6	24.6
Second grass-legume mix baleage	24.2	19.2
First cut grass-legume mix baleage	18.0	18.0
*C. crispus*	0.0	6.0
**Nutrient composition, % of DM unless otherwise noted**		
DM, % of fresh matter	77.3	78.0
Organic matter	90.5	88.8
Crude protein	16.9	16.7
Neutral detergent fiber	36.2	35.6
Acid detergent fiber	24.1	22.7
Acid detergent lignin	4.20	3.98
Ether extract	3.58	3.52
Ca	0.64	0.84
P	0.32	0.31

**Table 4:** Ingredient composition of the concentrate pellet fed during the animal case study feeding trial.

Ingredient	% of pellet (as fed basis)
Ground corn	39.2
Extruded soybean	16.5
Wheat middlings	13.0
Roasted soybean	7.99
Mixed grains^1^	6.99
Barley meal	6.99
Molasses	2.98
Salt	1.49
Sodium bicarbonate	1.45
Dicalcium phosphate	0.75
Mineral and vitamins premix^2^	0.67
Pellet binder	0.60
Magnesium oxide	0.49
Magnesium sulfate	0.33
Limestone	0.32
XPC yeast^3^	0.17
Zinpro 100^4^	0.06

1 Rye, wheat and peas (Morrison’s Custom Feeds, Barnet, VT).

2 Mineral and vitamin mix contained (as-fed basis): 8% Ca, 3.5% P, 16% salt, 8% Mg, 22 mg/kg of Co, 452 mg/kg of Cu, 1,162 mg/kg of Mn, 28.5 mg/kg of Se, 2,332 mg/kg of Zn, 220.3 kIU/kg of vitamin A, 22.0 kIU/kg of vitamin D, and 2.64 kIU/kg of vitamin E.

3 Fermented product generated from yeast cultures (Diamond V, Cedar Rapids, IA).

4 Feed ingredient containing 10% organic zinc (Zinpro, Eden Prairie, MN).

**Table 5:** Nutrient profile of dietary ingredients [% of dry matter (DM) unless otherwise noted].

	Baleage			Grain pellet	*Chondrus crispus*
Nutrient composition	First cut grass-legume mix	Second cut grass-legume mix	Alfalfa		
DM, % of fresh matter	68.1	70.9	76.4	87.5	86.1
Crude protein (CP)	10.3	15.4	20.5	18.9	13.0
Neutral detergent fiber (NDF)	57.3	54.2	34.2	13.0	38.6
Acid detergent fiber	36.2	36.8	28.2	5.30	7.90
Acid detergent lignin	4.20	5.90	5.72	1.70	2.30
Non-fiber carbohydrates (NFC)^1^	22.2	16.8	33.3	54.2	7.70
Ether extract (EE)	2.80	3.00	2.63	5.12	2.47
Ash	7.51	10.6	11.1	8.74	38.3
Ca	0.41	0.94	NA	1.02	4.27
P	0.27	0.33	NA	0.58	0.24
Mg	0.18	0.23	NA	0.59	0.95
K	1.79	2.15	NA	1.07	2.44
Na	0.12	0.12	NA	1.09	4.43
S	0.18	0.19	NA	0.31	5.51
Cl	0.58	0.61	NA	NA	NA
I, mg/kg	NA^2^	NA	NA	NA	404

1 NFC = 100 – (CP% + NDF% + EE% + ash%).

2 Not analyzed.

In the present animal case study feeding trial, individual DMI was not measured due to constraints in farm facilities. Alternatively, we estimated an average DMI of 20.6 and 19.6 kg/d for 0CC and 6CC, respectively, using an empiric model (Table 6). The decline in estimated DMI resulting from feeding 6CC is challenging to interpret due to the limitations of the model. However, a study conducted at the University of New Hampshire in which organic certified Jersey cows were fed incremental amounts of *C. crispus* (0, 3, and 6% diet DM basis) showed a linear decrease in DMI (20.7, 19.3, and 18.9 kg/d for 0, 3, and 6%, respectively), potentially associated with palatability issues (17). A similar response was observed when the red seaweed *A. taxiformis* was fed to lactating dairy cows (2).

**Table 6:** Emissions of carbon dioxide (CO_2_) and enteric methane (CH_4_)^1^, estimated dry matter intake (DMI), milk yield and composition, and body weight (BW) in lactating dairy cows supplemented with *Chondrus crispus* at 6% diet dry matter (6CC) or without (0CC).

	Treatment			*p*-value		
Item	0CC	6CC	SEM	Treatment (T)	Week (W)	T x W
Estimated DMI, kg/d^2^	20.6	19.6	-	-	-	-
Milk yield, kg/d	27.3	26.9	0.65	0.56	<0.01	0.04
4% FCM,^3^ kg/d	27.0	27.0	0.92	0.99	0.06	0.80
ECM,^4^ kg/d	29.4	29.4	0.94	0.95	0.05	0.85
Milk fat, %	3.89	3.79	0.01	0.42	0.35	0.57
Milk fat, kg/d	1.07	1.06	0.04	0.76	0.30	0.49
Milk true protein, %	3.10	3.06	0.04	0.33	0.05	0.87
Milk true protein, kg/d	0.86	0.86	0.02	0.97	0.004	0.76
CH_4_, g/d	466	401	10.2	<0.001	0.57	0.72
CO_2_, kg/d	12.1	11.5	0.26	0.11	0.27	0.74
CH_4_, g/kg of ECM	15.9	14.5	0.61	0.11	0.09	0.67
BW, kg	480	495	8.91	0.08	0.31	0.89

1 Gases were measured using 1 GreenFeed unit (C-Lock Technology Inc., Rapid City, SD). The average number of visits to the GreenFeed unit was 23 visits/cow during each sampling week, with an average of 05:24 min/visit, and 3.5 ± 1.48 visits/day/cow.

2 Estimated DMI, kg/d = (0.372 × 4% FCM (kg/d) + 0.0968 × BW^0.75^) × {1–e^[−0.192 × (Week of Lactation + 3.67)]^} (20).

3 4% FCM (fat corrected milk) = (0.4 × kg of milk) + (15 × kg of milk fat) (22).

4 ECM (energy corrected milk) = (0.327 × kg of milk) + (12.95 × kg of milk fat) + (7.65 × kg of milk protein) (23).

**Figure 1:**
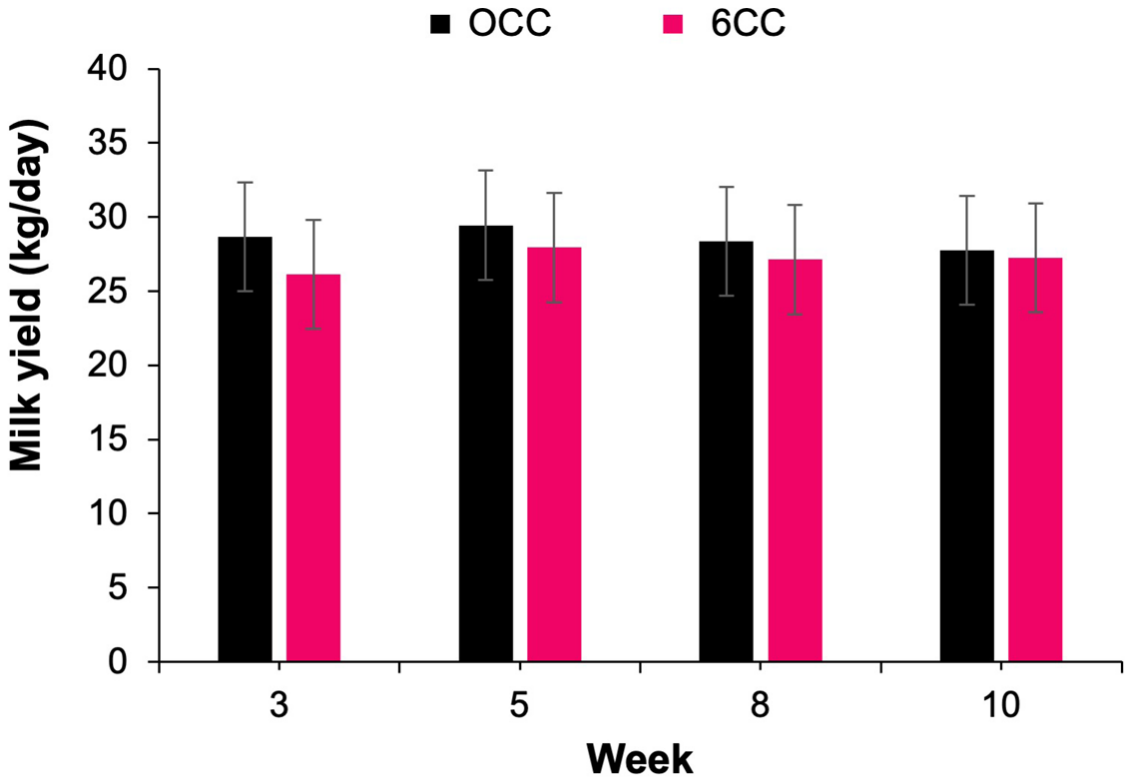
Weekly milk yield in cows supplemented either 0 or 6% dry matter of the red seaweed *Chondrus crispus*. Treatment effect, *p* = 0.55; week effect, *p* < 0.001; interaction of treatment by week, *p* = 0.04.

**Figure 2:**
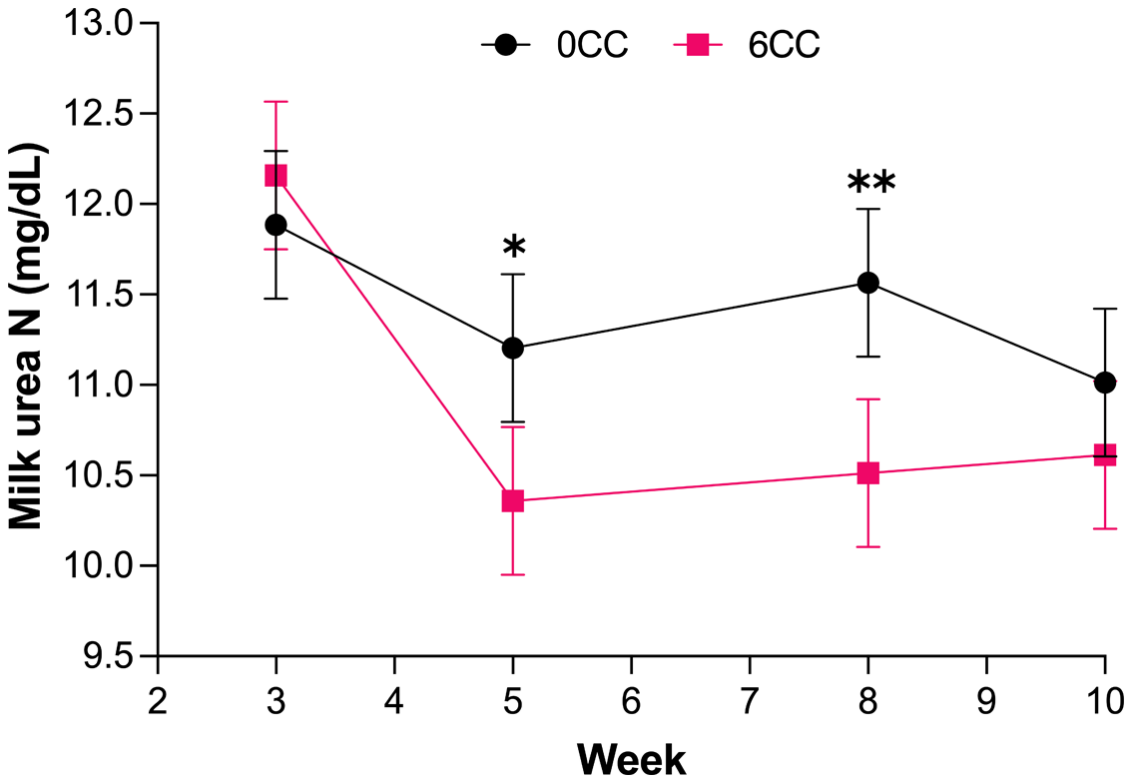
Weekly milk urea N concentration in cows supplemented either 0 or 6% dry matter of the red seaweed *Chondrus crispus*. Treatment effect, *p* = 0.35; week effect, *p* < 0.001; interaction of treatment by week, *p* = 0.05. *Indicates statistical tendency (*p* = 0.1) and **difference between treatments (*p* = 0.04).

A treatment by week interaction (*p* = 0.04) was observed for milk production (Table 6), but treatments did not differ from each other during weeks 3, 5, 8, and 10 (Figure 1), suggesting that the effect of lactation progression was different for the 2 treatments. Likewise, a treatment by week interaction was detected for MUN concentration (*p* = 0.05; Figure 2). Cows in 6CC treatment had lower (*p* = 0.04) MUN concentration than those in the 0CC treatment during wk. 8 (11.6 vs. 10.5 mg/dL), and a tendency (*p* = 0.10) for reduced MUN concentration during wk. 5 (11.2 vs. 10.4 mg/dL). It is well known that dietary CP concentration is the main driver of MUN (36). However, dietary CP concentration was similar between treatments (Table 4) and likely did not account for the observed decrease in MUN with feeding 6CC on weeks 5 and 8. Concentrations and yields of milk fat and milk true protein, as well as 4% FCM and ECM yields were not affected (*p* > 0.05) by treatments possibly because the difference in estimated DMI was small when comparing 0CC versus 6CC diets (Table 6). The observed lack of effect of *C. crispus* on lactational performance in the current study is consistent with our recent work (17).

Enteric CH_4_ production decreased by 13.9% in dairy cows fed the 6CC (401 g/d) versus the 0CC (466 g CH_4_/d) as shown in Table 6. This response surpassed the 8.4% reduction in enteric CH_4_ production (from 383 to 351 g CH_4_/d) observed in our previous research in which Jersey cows were fed incremental amounts (0, 3, and 6% of the diet DM) of *C. crispus* (17). Such discrepancy in response magnitude between our 2 studies may be explained by using different batches of *C. crispus*, as well as differences in the basal diets, dairy breed, management practices, and concentration of antimethanogenic compounds present in *C. crispus* tissues. In our recent study (17), the linear decline in enteric CH_4_ production was accompanied by a linear reduction in DMI as *C. crispus* inclusion increased in the diet DM. The positive correlation between DMI and enteric CH_4_ production is well established in the literature (19). Estimated DMI was 4.9% lower with feeding 6CC versus 0CC in the current experiment, which may have contributed to the observed drop in CH_4_ production. However, DMI was not individually measured herein and estimated DMI data should be interpreted cautiously, particularly because the equation (20) used to calculate DMI does not account for specific diet nutrient characteristics. Both CO_2_ production (mean = 11.8 kg/d), and CH_4_ intensity (mean = 15.2 g of CH_4_/kg of ECM) did not differ between treatments, thus in line with our recent research (17).

Certain red seaweeds have been reported for having high antimethanogenic activity *in vitro* (37, 38) and *in vivo* (2, 7). For instance, dietary supplementation with *A. armata* and *A. taxiformis* at 1 and 0.5% (organic matter basis, respectively) effectively suppressed enteric CH_4_ production in dairy cows by 67.2 and 34.4%, respectively, relative to a control diet (0% seaweed inclusion) (2, 7). These responses were attributed to the presence of halogenated or brominated compounds (e.g., bromoform) that bioaccumulate in the tissues of some red seaweeds (4). A recent genomic sequencing reported that *C. crispus* possesses the enzymes required to synthesize halogenated compounds (39). However, in the present study, none of the *C. crispus* samples tested had detectable levels of bromoform, nor any other brominated metabolites. Thus, it is possible that unknown bioactive compounds may be involved and should be identified to better understand the potential of *C. crispus* to decrease enteric CH_4_ emissions in dairy cows. By extrapolating the 13.9% reduction in CH_4_ production from this feeding trial to the current size of Maine’s organic dairy sector and average estimates of annual CH_4_ emitted per cow [100 kg/cow (40)], we estimate that nearly 50,000 kg of CH_4_ originating from 3,600 cows would be suppressed each year. At current estimates of the social cost of CH_4_ [i.e., 1,756 USD/ton of CH_4_ emitted (41)], this is an annual external benefit of over 87,000 USD from Maine organic dairies alone. Nonetheless, to effectively engage and maximize widespread adoption of algal-based feeds by producers, it may be necessary to implement subsidies or participation in carbon offset markets when available.

## 4. Conclusion

There has been much discussion regarding the utilization of seaweeds in dairy cattle diets. This study surveyed Maine’s organic dairy producers for their willingness to adopt an antimethanogenic algal-based feed. About 93% of organic dairy producers in our survey cited deteriorating infrastructure, rising costs, and instability in the supply chain as pressing concerns before they mentioned climate change and climate regulation. Therefore, producer’s receptiveness to implement seaweeds in dairy diets will hinge not only on costs but also on the documentation of co-benefits, government policies and subsidies, and the ease of integration into existing feeding management practices. Concurrently, we conducted an animal case study feeding trial to evaluate the antimethanogenic potential of the red seaweed *C. crispus* in a typical Maine organic dairy farm. Feeding *C. crispus* at 6% of the diet DM decreased enteric CH_4_ production in dairy cows by 13.9% with no adverse effect on milk production and composition. Further research exploring *C. crispus* bioactive compounds will help to better understand the effect of this red seaweed on mitigating enteric CH_4_ emissions in ruminants.

## Data availability statement

The raw data supporting the conclusions of this article will be made available by the authors, without undue reservation.

## Ethics statement

The studies involving human participants were reviewed and approved by Colby College Institutional Review Board. The patients/participants provided their written informed consent to participate in this study. The animal study was reviewed and approved by University of New Hampshire Institutional Animal Care and Use Committee.

## Author contributions

AB, CQ, and NP contributed to conception and design of study. JM and MD designed and implemented the producer survey. DR, LP, KB, RK, and CQ carried out the animal feed trial, taking samples, feeding animals, etc. DR and AB performed the statistical analysis of the animal feed trial data and JM and MD of the survey. KS and DC contributed with facilities/equipment. DR and JM wrote the first draft of the manuscript. All authors contributed to the article and approved the submitted version.

## Funding

This work was supported by the Shelby Cullom Davis Charitable Fund and the Sustainable Agriculture Systems grant no. 2021-69012-35919 from the USDA National Institute of Food and Agriculture. Partial funding was provided by the New Hampshire Agricultural Experiment Station (Durham, NH; Scientific Contribution Number 2985). This work was further supported by the USDA-National Institute of Food and Agriculture Hatch Multistate NC-2042 (Project Number NH00670-R; Kansas City, MO).

## Conflict of interest

The authors declare that the research was conducted in the absence of any commercial or financial relationships that could be construed as a potential conflict of interest.

## Publisher’s note

All claims expressed in this article are solely those of the authors and do not necessarily represent those of their affiliated organizations, or those of the publisher, the editors and the reviewers. Any product that may be evaluated in this article, or claim that may be made by its manufacturer, is not guaranteed or endorsed by the publisher.
