# Supplementary material for: Maine organic dairy producers’ receptiveness to seaweed supplementation and effect of Chondrus crispus on enteric methane emissions in lactating cows
Source: Front Vet Sci. 2023 Jul 6;10:1153097. doi: 10.3389/fvets.2023.1153097 (PMC10356979; doi:10.3389/fvets.2023.1153097)
Supplement: Supplementary file 1 [file Data_Sheet_1.docx]

**Appendix A: Initial Maine Organic Dairy Respondent Flyer/Primer**

**Appendix B: Maine Organic Dairy Farmer Survey Questions**

Dairy Farmer Survey

Start of Block: Block 1

*Hello!  My name is                                  and I’m a student at Colby College following up on an invitation you received from Rick Kersbergen to participate in a confidential survey of organic dairy farmers. about seaweed grain supplements.*
*Did you get a chance to take a look at the info-graphic that came with Rick's invitation?*
**If “no” then:**  "That's okay, I can explain or answer any questions you might have as we do the survey together. The most important thing for you to know is that this is an anonymous survey.  None of your answers will be linked to you or your farm."
*Do you have about 15 minutes to participate in this project?***If “no” then:***"When would be a good time for our team to contact you?"*                If “not sure” or “don’t have time to talk” then:
*"Could I send you a copy of the survey?"*                               If “no” then: *"All right, I understand.  I hope that you stay safe and have a nice day."*                (if they give a reason why they don’t want to participate then record it)                                If “yes” then: *"Would you prefer a hard copy of the survey or a link to do it online?"*                                     Get email address if they prefer a link **If offered a time to reschedule then**: *"Okay, I’ve got you on our schedule and either I or someone from our team here at Colby will call you then. Thank you very much."* **If “yes” then:** *"Okay, let’s get started."*If they ask for what the results of the survey will be used for, then: *"The results of this survey will also help us determine the potential for seaweed supplements to be a cost effective part of dairy farming operations like yours."*

Q38 Reason for not participating (if applicable)

________________________________________________________________

Q3 How many cattle are you milking?

- Less than 50 (1)
- 50 - 100 (2)
- 101 - 150 (3)
- 151 - 200 (4)
- More than 200 (5)
- Refuse to Answer (6) __________________________________________________
- Enter a Specific Number (7) __________________________________________________

Q4 What is your average production per cow per day? (pounds)

- Less than 30 (1)
- 30-54 (2)
- 55-74 (3)
- 75-94 (4)
- 95-115 (5)
- More than 115 (6)
- Refuse to Answer (7) __________________________________________________
- Enter a Specific Number (8) __________________________________________________

Q41 What is the average % butterfat of your milk?

________________________________________________________________

Q42 What is the average % protein of your milk?

________________________________________________________________

Q43 What is the average Somatic Cell Count?

________________________________________________________________

Q5 How much milk did you ship in 2019? (CWT) 


*The farmer may give a number in lbs. so remove two decimal points to convert lbs to CWT, e.g., 100,000 pounds is 1,000 "hundred-weight" (CWT)*

________________________________________________________________

Q6 How much more (or less) to you expect to ship this year? (% or CWT)

________________________________________________________________

Q7 What range best fits your average hundred-weight contract price of your milk? (base price)

- $20 - $25 (1)
- $26 - $30 (2)
- $31 - $35 (3)
- $36 - $40 (4)
- More than $40 (5)
- Refuse to Answer (6) __________________________________________________
- Enter a Specific Number (7) __________________________________________________

Q8 Who do you ship your milk to? (Check all that apply)

- Stonyfield (1)
- Organic Valley (CROPP) (2)
- Horizon Milk (3)
- Other (4) __________________________________________________
- Refuse to Answer (5) __________________________________________________

Q9 Do you sell milk to any other places? (check all that apply)

- None (6)
- Processors (cheese, yogurt, and other milk products) (1)
- Direct to retail stores (e.g grocery stores or natural food stores) (2)
- Direct to consumers (e.g., customer pickup, coops/farm shares, farmers markets) (3)
- Other sales (4) __________________________________________________
- Refuse to Answer (5) __________________________________________________

End of Block: Block 1

Start of Block: Block 3

Feeding Practices *Next, we'd like learn about your feeding practices*

Q11 During the months of the year when pasture is available, what percentage of your dairy cows' diet is pasture in terms of dry matter?

________________________________________________________________

Q40
During the months of the year when pasture is available, how often do you feed your cows grain per day? (Note: Farmers may say TMR - Total Mixed Ration - which means that they put a mix in front of the cows and they have access to it throughout the day)

- Twice a day (4)
- Once a day (5)
- TMR (6)
- Other (7) __________________________________________________

Q12
During the months of the year when pasture is available, how much grain do you feed your cattle per day? (lbs / cow) [Note: some farmers may distinguish between 'high producing' cattle and 'low producers' - record everything]

________________________________________________________________

Q47 During the months of the year when pasture is NOT available, how often do you feed your cows grain per day? (Farmers may mention TMR in their answer here. TMR is Total Mixed Ration, a method of feeding dairy cattle. The purpose of feeding a TMR diet is that each cow can consume the required level of nutrients in each bite. A cow's ration should include good quality forages, a balance of grains and proteins, vitamins and minerals.
day)

- Twice a day (1)
- Once a day (2)
- Freely available (3)
- Other (4) __________________________________________________

Q50 During the months of the year when pasture is NOT available, how much grain do you feed your cattle per day? (lbs / cow) [Note: some farmers may distinguish between 'high producing' cattle and 'low producers' - record everything]

________________________________________________________________

Q13 Where do you get your grain from?

________________________________________________________________

Q51 What form is the grain in?

- Grain meal (1)
- Pellet (2)
- Other (4) __________________________________________________

Q49
What are the components and approximate percentages in your grain mix? (If the farmer says "not sure" or doesn't have the information available, ask if we can contact their feed supplier or if they could send us the formula for their gain mix).

- Not sure (1)
- Item 1 (e.g., corn - 25%) (2) __________________________________________________
- Item 2 (3) __________________________________________________
- Item 3 (4) __________________________________________________
- Item 4 (5) __________________________________________________
- Item 5 (6) __________________________________________________
- Item 6 (7) __________________________________________________
- Item 7 (8) __________________________________________________
- Item 8 (9) __________________________________________________
- Item 9 (10) __________________________________________________
- Additional items (11) __________________________________________________

Q14 What is your approximate cost per ton of grain?

________________________________________________________________

Q15 Beyond the minerals and vitamins in your grain mix, do you feed other supplements to your dairy cattle? (check all that apply)

- None (1)
- Yeast (2)
- Sodium Bicarbonate (4)
- Additional vitamins & minerals (5)
- Salt (6)
- Other (7) __________________________________________________
- Refuse to Answer (8) __________________________________________________

Skip To: End of Block If Beyond the minerals and vitamins in your grain mix, do you feed other supplements to your dairy c... = None

Skip To: End of Block If Beyond the minerals and vitamins in your grain mix, do you feed other supplements to your dairy c... = Refuse to Answer

Q16 What is the most important nutrient, beyond protein or energy levels, in choosing your supplements during the grazing season?

- Vitamins (1)
- Macro minerals such as Calcium and Phosphorus (2)
- Micro nutrients such as Selenium (3)
- Digestion aides such as sodium bicarbonate or probiotics (4)
- Other (5) __________________________________________________
- Refuse to Answer (6) __________________________________________________

Q17 What is the approximate cost per cow of these additional nutritional supplements?

________________________________________________________________

End of Block: Block 3

Start of Block: Block 2

Final *In this final set of questions, we'd like to get your feedback on how a seaweed grain supplement might fit into your operations.*

Q19 Do you feed your dairy cattle a seaweed or kelp supplement?

- No (Enter why) (1) __________________________________________________
- SeaLife Kelp Meal (4)
- Thorvin Kelp (5)
- Acadian (6)
- Other (7) __________________________________________________

Skip To: Q44 If Do you feed your dairy cattle a seaweed or kelp supplement? = No (Enter why)

Q41 What is the primary reason you feed it or don't feed it?

________________________________________________________________

Q43 How much of this seaweed supplement do you feed per cow?

- Enter amount: (1) __________________________________________________
- Not sure (2)
- Refused to answer (enter why) (3) __________________________________________________

Q42 How do you feed the seaweed supplement to your cattle?

- Mixed with the grain (1)
- Top dress (2)
- Other (4) __________________________________________________

Q44 Do you know how much more it costs per cow to feed a seaweed supplement?

- Enter amount: (1) __________________________________________________
- Not sure (2) __________________________________________________
- Refused to answer (enter why) (3) __________________________________________________

Q20 If different types of seaweed supplements become available that would reduce the amount of methane emissions from your cows would you be interested in adding it to their feed?

- Yes (1)
- Maybe (3) __________________________________________________
- No (please explain) (2) __________________________________________________

Q23 How much of an increase in your daily feed costs per cow would you be willing to pay for a seaweed supplement that also reduced methane emissions?

- Amount (1) __________________________________________________
- Not sure or "it depends" *(Ask what factors would influence how much you would be willing to pay for a seaweed grain supplement)* (2) __________________________________________________
- Refuse to Answer (3) __________________________________________________

Q27 Because methane can also come from manure it's possible that seaweed grain supplements may have an impact on your manure management strategies. What are your current manure management practices? (check all that apply)

- None (7)
- Liquid manure pit (1)
- Solid manure stacking site (2)
- Field stacking (3)
- Bedded pack (8)
- Composting (4)
- Other: (5) __________________________________________________
- Refuse to Answer (6) __________________________________________________

Q28 What are the top 3 challenges you face as an organic dairy farmer in Maine?

Q31 First Challenge

________________________________________________________________

Q35 Second Challenge

________________________________________________________________

Q36 Third Challenge

________________________________________________________________

Q29 *We’ve worked with the Institutional Review Board at Colby College to ensure that all of your answers will be treated confidentially and will not be shared or published in any fashion that might identify you or your operations beyond the research team at Colby College.*
Would if be okay if we followed up with you later in our project to get your advice or hear more about your experiences once we've begun our feeding trials?

- Yes (1)
- No (2)

Display This Question:

If We’ve worked with the Institutional Review Board at Colby College to ensure that all of your answ... = Yes

Q44 What would be the best way for us to follow up?

- Phone call (4)
- U.S. Mail (5)
- Email (6) __________________________________________________
- Other (7) __________________________________________________

Q39 *Thank you very much for your time.  I enjoyed talking with you and learned a lot. This has been extremely helpful.*

Q52 Status of survey

- Complete (1)
- Incomplete - respondent did not want to, or was unable to, finish (2)
- Incomplete - rescheduled for: (3) __________________________________________________

Q53 Your name (student who administered this survey)

________________________________________________________________

Q54 Database ID of farmer

________________________________________________________________

End of Block: Block 2

Appendix C: Summary Statistics of Maine Organic Dairy Farmer Survey

Table C.1

|  |  | |  | | |
| --- | --- | --- | --- | --- | --- |
| Variable | Observations | Mean | Std Dev | Min | Max |
| Cows per herd | 35 | 53.29 | 47.1 | 5 | 225 |
| Milk yield, kg/cow/day | 34 | 21.09 | 6.96 | 5.44 | 33.57 |
| Milk fat, % | 35 | 4.34 | 0.55 | 3.4 | 6 |
| Milk true protein, % | 32 | 3.89 | 1.03 | 2.5 | 8.75 |
| Somatic cell count, (×10**^3^** cells/mL) | 35 | 123.3 | 50.3 | 20 | 284 |
| Milk shipped, kg/yr | 32 | 488,755 | 506,664 | 0 | 2,032,094 |
| Contract price, USD/cwt of milk^3^ | 33 | 31.01 | 7.02 | 7 | 52.32 |
| Pasture diet, % | 32 | 65.25 | 21.65 | 15 | 100 |
| Grain cost, USD/ton | 33 | 613 | 75.75 | 490 | 818 |
| Willingness to pay, USD/cow/day | 35 | 0.64 | 1.33 | 0 | 5 |

^3^Cwt = milk price received per hundredweight (cwt = 45.36 kg)
